# Comparing pyrotinib with trastuzumab and pertuzumab with trastuzumab for HER2-positive metastatic breast cancer: a retrospective, multicenter analysis

**DOI:** 10.3389/fendo.2023.1325540

**Published:** 2023-12-11

**Authors:** Shuhui You, Yizhao Xie, Die Sang, Ting Luo, Peng Yuan, Fei Xu, Biyun Wang

**Affiliations:** ^1^ Department of Breast and Urological Medical Oncology, Fudan University Shanghai Cancer Center, Shanghai, China; ^2^ Department of Oncology, Shanghai Medical College, Fudan University, Shanghai, China; ^3^ Department of Medical Oncology, Zhongshan Hospital, Fudan University, Shanghai, China; ^4^ Department of Medical Oncology, Sanhuan Cancer Hospital, Beijing, China; ^5^ Department of Head, Neck and Mammary Gland Oncology, Cancer Center, West China Hospital, Sichuan University, Sichuan, China; ^6^ National Cancer Center, Tumor Hospital of the Chinese Academy of Medical Sciences, Beijing, China; ^7^ Department of Medical Oncology, Sun Yat-Sen University Cancer Center, The State Key Laboratory of Oncology in South China, Collaborative Innovation Center for Cancer Medicine, Guangzhou, Guangdong, China

**Keywords:** human epidermal growth factor receptor 2 (HER2), metastatic breast cancer (MBC), pyrotinib, trastuzumab, pertuzumab

## Abstract

**Objective:**

Pyrotinib and pertuzumab are effective treatment options for HER2-positive metastatic breast cancer (HER2+ MBC). Our study was to directly compare the efficacy and safety of pyrotinib plus trastuzumab (PyroH) and pertuzumab plus trastuzumab (HP) in patients with HER2+ MBC.

**Methods:**

We conducted a retrospective examination of HER2+ MBC patients who received PyroH plus chemotherapy or HP plus chemotherapy between 2017 and 2022 at five institutions in China. Our primary endpoint was progression-free survival (PFS).

**Results:**

This study involved 333 patients, among which 161 received PyroH and 172 received HP. The utilization of PyroH as a first-line therapy for MBC was more prevalent among older patients, those with a shorter duration of disease-free interval, or those who had previously been treated with trastuzumab. Although in the first-line advanced treatment HP cohort showed numerically longer PFS (median PFS: 14.46 vs. 22.90 months, *p*=0.057), in the second-line or later treatments, there was no significant difference in PFS between the PyroH and HP groups (median PFS: 8.67 vs. 7.92 months, *p*=0.286). Despite HP showing a longer PFS in the overall cohort (median PFS: 9.30 vs. 13.01 months, *p*=0.005), it did not serve as an independent predictor of PFS in the multivariate analysis (HR 1.134, 95% CI 0.710-1.811, p=0.598). Without taxane, PyroH demonstrated a longer PFS than HP (median PFS: 10.12 vs. 8.15 months, *p*=0.017). PyroH group displayed a numerically longer median PFS in patients with brain metastases compared to the HP group, though not statistically significant (median PFS: 9.03 vs. 8.15 months, *p*=0.976). PyroH had higher incidence of grade 3/4 diarrhea (34.3% vs. 3.0%) but similar overall adverse events.

**Conclusion:**

In conclusion, PyroH is comparable in second-line or later treatment and during brain metastasis, even having superior efficacy without taxane in real-world setting. Toxicities were tolerable in both groups. (ClinicalTrials.gov: NCT05572645)

## Introduction

Breast cancer stands as the predominant cancer among women globally, accounting for a significant proportion of cancer-related mortalities (1). Around 15-20% of these breast cancer patients show an overexpression of human epidermal growth factor receptor 2 (HER2), linked with a more aggressive progression and inferior prognosis (2).

The medical landscape for HER2-positive metastatic breast cancer (HER2+ MBC) has considerably evolved over the past few decades, driven by the advent of therapeutics targeting HER2, which primarily includes monoclonal antibodies, tyrosine kinase inhibitors (TKIs), and antibody-drug conjugates (ADCs).

Pertuzumab, in combination with trastuzumab (HP) and docetaxel, is considered the standard first-line dual anti-HER2 therapy for HER+ MBC (3). Meanwhile, pyrotinib (Pyro) is an irreversible pan-HER receptor small-molecule tyrosine kinase inhibitor (4), confirming the efficacy of the pyrotinib regimen in second- and post second-line treatment of HER2+ MBC (5–7). The phase III PHILA trial demonstrated that adding pyrotinib to trastuzumab (PyroH) and docetaxel markedly extends progression-free survival (PFS) in first-line treated HER2+ MBC patients, as opposed to the combination of placebo, trastuzumab, and docetaxel (median PFS: 24.3 vs. 10.4 months, p< 0.0001) (8). Dual-target therapy combining large and small molecules offers a promising approach for the HER2+ MBC management. However, no direct comparison has been made between PyroH and HP in this patient population.

Hence, the aim of this multicenter, retrospective study is to compare the therapeutic patterns, efficacy, and safety profiles of PyroH and HP regimens for HER2+ MBC patients in real-world clinical practice.

## Methods

### Patients and treatments

We conducted a search across the databases of five prestigious institutions, namely the Fudan University Shanghai Cancer Center, Sun Yat-sen University Cancer Center, Beijing San Huan Cancer Hospital, West China Hospital Sichuan University, and Tumor Hospital of the Chinese Academy of Medical Sciences, to identify all patients with HER2+ MBC who received PyroH or HP between June 2017 and December 2022.

For inclusion in this study, participants needed to satisfy the following prerequisites: (1) confirmed pathological diagnosis of HER2+ MBC, ascertained by either a +3 score through immunohistochemistry (IHC) analysis or a +2 score coupled with a positive fluorescence *in situ* hybridization (FISH) result; (2) receipt of treatment involving pyrotinib (320-400 mg, orally, daily) in combination with trastuzumab (initially 8 mg/kg every three weeks, followed by 6 mg/kg every three weeks) and chemotherapy, or pertuzumab (initially 840mg every three weeks, followed by 420 mg every three weeks) in combination with trastuzumab (initially 8 mg/kg every three weeks, followed by 6 mg/kg every three weeks) and chemotherapy for at least one cycle, between June 2017 and December 2022; (3) comprehensive medical records available in each institution’s electronic medical record system. Exclusion criteria included incomplete medical history and concurrent use of pyrotinib and pertuzumab in the same treatment regimen.

This research was sanctioned by the Fudan University Shanghai Cancer Center’s Ethics Committee and Institutional Review Board (SCCIRB, 1812195-6), in accordance with the Declaration of Helsinki and applicable guidelines. It’s also recorded on clinicaltrials.gov (NCT 05572645).

### Measurement of outcomes

Our study’s primary outcome was the duration of PFS, complemented by overall survival (OS) and safety as secondary outcomes. PFS was defined as the duration from the start of treatment to either disease progression or death from any cause. OS marked the interval from the onset of treatment to any-cause death. Safety evaluations were conducted based on version 4.03 of the National Cancer Institute’s Common Terminology Criteria for Adverse Events (CTCAE). Tumor responses were determined according to the RECIST 1.1 standards. The definition of disease-free interval (DFI) was the time from primary surgery to the diagnosis of MBC. Resistance to trastuzumab was characterized by the emergence of fresh relapses during or within a year after adjuvant trastuzumab or the disease progression within three months following first-line trastuzumab treatment in the metastatic stage (9). Refractoriness of trastuzumab was characterized as the development of disease progression after receiving two or more trastuzumab-based treatment lines, which initially resulted in disease response or stabilization at the initial radiological evaluation (9).

### Statistics

The study encompassed all qualifying patients for evaluation. Clinicopathological factors were captured using descriptive statistical methods, and the Chi-square test was implemented for group comparison. PFS and OS were assessed through the Kaplan-Meier approach, while hazard ratios (HRs) and corresponding 95% confidence intervals (95% CIs) were assessed via the Cox proportional hazards framework. Log-rank tests were conducted for the comparison of survival distributions across the study groups. Forest plots based on the Cox regression model were used for subgroup analysis. The connection between predictor variables and the time-to-event outcome was examined utilizing univariate and multivariate Cox proportional hazards models through SPSS version 25. P-values below 0.05 were recognized as having statistical significance. Graphpad Prism version 8.0 was utilized to generate Kaplan-Meier curves and forest plots.

## Results

### Baseline characteristics

This study included 333 patients, among which 161 received PyroH and 172 received HP. The comparison of their baseline characteristics was presented in **Table 1**. The distribution of pathological grades was comparable between the two groups (*p*=0.153), and the DFI exhibited no significant divergence (*p*=0.341). However, fewer patients in the PyroH group were diagnosed *de novo* stage IV breast cancer (26.1% vs. 41.9%, p=0.006) and more had multiple metastatic sites (53.4% vs 45.9%, p=0.032). The occurrence of brain metastasis was markedly higher in the PyroH cohort (35.4% vs. 17.4%, p<0.001). The proportion of trastuzumab-refractory patients was substantially larger in the PyroH group (65.8% vs. 36.6%) while the rate of trastuzumab-sensitivity was lower (11.2% vs. 48.3%) (p<0.001). Previous metastatic disease treatment lines and trastuzumab therapy also differed between groups (*p*<0.001). The two groups had similar baseline characteristics, but disease severity and treatment history differed. Thus, these disparities should be considered when comparing treatment outcomes between groups.

**Table 1:** Baseline characteristics of patients grouped by PyroH or HP.

	Pyrotinib plus trastuzumab group (PyroH) N=161 n (%)	Pertuzumab plus trastuzumab group (HP) N=172 n (%)		p-value
Median age (range), years	51 (25–83)	51 (25–71)		0.212
Eastern Cooperative Oncology Group performance status				0.669
0	15 (9.3)	19 (11.0)		
1	120 (74.5)	134 (77.9)		
2	11 (6.8)	7 (4.1)		
3	3 (1.9)	4 (2.3)		
Hormone receptor status				0.326
Oestrogen receptor or progesterone receptor positive	89 (55.3)	87 (50.6)		
Oestrogen receptor and progesterone receptor negative	70 (43.5)	85 (49.4)		
Pathological grading				0.153
I	4 (2.5)	0 (0.0)		
II	50 (31.1)	44 (25.6)		
III	59 (36.6)	57 (33.1)		
Unknown	48 (29.8)	60 (34.9)		
Disease-free interval				0.341
<2years	56 (34.8)	40 (23.3)		
≥2years	63 (39.1)	59 (34.3)		
*de novo* stage IV breast cancer	42 (26.1)	72 (41.9)		0.006
Number of metastatic sites				0.032
1	24 (14.9)	46 (26.7)		
2	50 (31.1)	47 (27.3)		
≥3	86 (53.4)	79 (45.9)		
Metastatic sites at screening				
Visceral	131 (81.3)	131 (76.2)		0.202
Brain	57 (35.4)	30 (17.4)		<0.001
Trastuzumab resistance status				<0.001
Resistance	37 (23.0)	24 (14.0)		
Refractoriness	106 (65.8)	63 (36.6)		
Sensitivity	18 (11.2)	83 (48.3)		
Previous treatment lines for metastatic disease				<0.001
0	24 (14.9)	99 (57.6)		
1	47 (29.2)	21 (12.2)		
≥2	89 (55.3)	52 (30.2)		
Previous trastuzumab therapy				<0.001
Yes	150 (90.0)	100 (58.1)		
No	11 (6.8)	71 (41.3)		
Combined with taxane				<0.001
Yes	41 (25.5)	137 (79.7)		
No	120 (74.5)	35 (20.3)		
Combined with capecitabine or vinorelbine or VP-16				<0.001
Yes	85 (52.8)	37 (21.5)		
No	76 (47.2)	135 (78.5)		

PyroH was given to 24 patients as the first-line systemic treatment for MBC, while HP was administered to 100 patients (**Supplementary Table 1**). PyroH was found to be more frequently utilized in older patients (*p*=0.004) and in those with a DFI of less than two years (37.5% vs. 11.0%, *p*=0.031) compared to HP during first-line treatment. 29.2% (7/24) of patients receiving PyroH and 12.0% (12/100) of patients receiving HP had brain metastases, although the difference was not significant (*p*=0.055) due to the small sample size. On first-line therapy, 16.7% (4/24) of PyroH patients were trastuzumab-resistant, compared to 3.0% (3/100) in the HP cohort, which had a higher proportion of trastuzumab-sensitive patients (HP: 80%, 80/100 vs. PyroH: 66.7%, 16/24) (*p*=0.078). 12/24 PyroH patients (50.0%) had previously received trastuzumab, compared to 22.0% (22/100) of HP patients (*p*=0.009). In summary, PyroH was used as a first-line therapy for MBC more frequently in older patients and those with a shorter DFI or previously treated with trastuzumab. There was a greater percentage of patients who were resistant to trastuzumab in the PyroH group, while the small sample size limited the statistical significance of some findings.

### Treatment patterns

**Figure 1:**
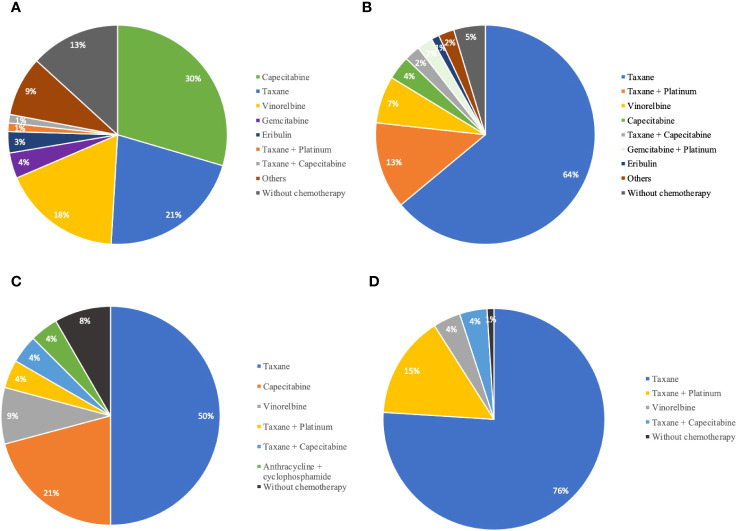
The distribution of chemotherapy agents in combination with PyroH and HP for MBC treatment. **(A)** The proportion of chemotherapy agents combined with PyroH in general MBC treatment. **(B)** The distribution of chemotherapy drugs used in conjunction with HP for general MBC treatment. **(C)** The distribution of chemotherapy agents used with PyroH in the context of first-line systemic therapy for MBC. **(D)** The proportion of chemotherapy drugs combined with HP in the scope of first-line systemic therapy for MBC.

HP was usually given with taxane-based chemotherapeutic agents (79.7%, 137/172), while PyroH was more often given with oral agents like capecitabine, vinorelbine, and VP-16 (52.8%, 85/161), which was statistically significant (*p*<0.001, **Table 1**). Capecitabine was the most frequently prescribed regimen among the 161 patients in the PyroH group (48/161, 29.8%), followed by taxane (34/161, 21.1%) and vinorelbine (28/161, 17.4%) (**Supplementary Table 2**, **Figure 1A**). In the HP group, taxane was the most commonly prescribed chemotherapy regimen (110/172, 64%), followed by platinum-based chemotherapy (26/172, 15.1%), including taxane + platinum, gemcitabine + platinum, and capecitabine (6/172, 3.5%) (**Supplementary Table 2**, **Figure 1B**). These findings indicate that PyroH and HP were administered with distinct chemotherapy regimens in clinical practice.

During first-line treatment, the HP group preferred a taxane-based chemotherapy combination (PyroH: 13/24, 54.2% vs. HP: 94/100, 94.0%, p<0.001, **Supplementary Table 3**). Taxane was the most common first-line systemic treatment for PyroH patients, followed by capecitabine (5/24, 20.8%) and vinorelbine (2/24, 8.3%) (**Supplementary Table 4**, **Figure 1C**). In the HP group, taxane was also the most common regimen, given to 76% (76/100) of patients, followed by taxane plus platinum (15/100, 15%), vinorelbine (4/100, 4%), and taxane plus capecitabine (4/100, 4%) (**Supplementary Table 3**, **Figure 1D**).

### Efficacy

**Figure 2:**
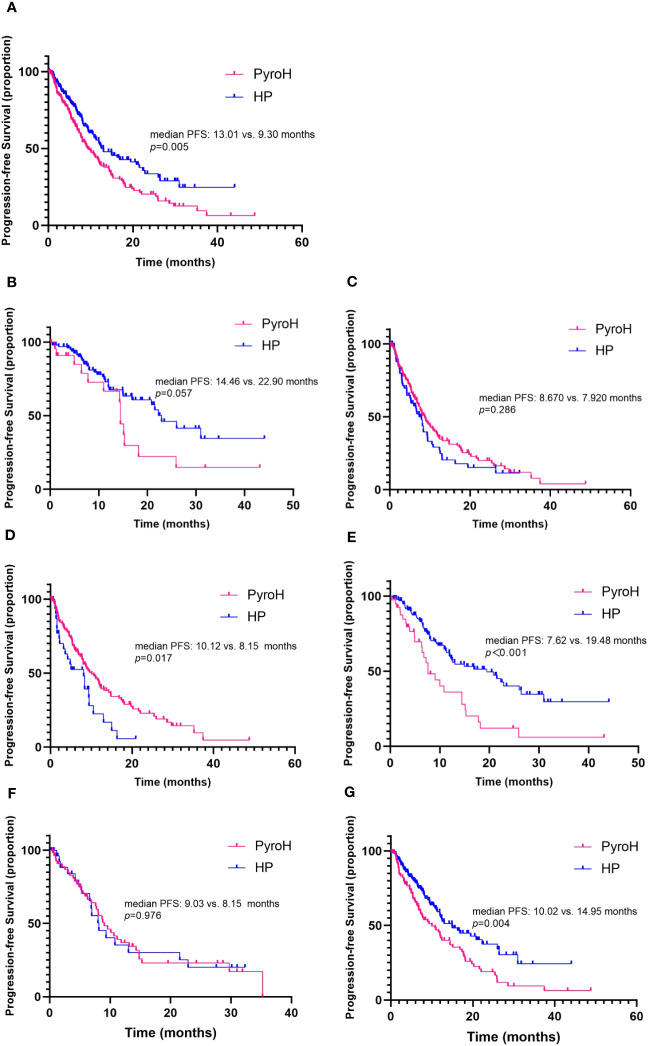
Kaplan-Meier curves illustrate progression-free survival, stratified by treatment arm. **(A)** The overall progression-free survival. **(B)** Progression-free survival in the context of first-line systemic treatment. **(C)** Progression-free survival for patients receiving second-and-later lines of systemic treatment for MBC. **(D, E)** Progression-free survival for patients combined without and with taxane respectively. **(F, G)** Progression-free survival for patients with and without brain metastases, respectively.

After a median follow-up period of 16 months, it was observed that 105 out of 161 patients in the PyroH cohort and 80 out of 172 patients in the HP cohort experienced progressive disease. Overall, PyroH exhibits a median PFS of 9.30 months, whereas HP displays a median PFS of 13.01 months (HR 1.51, 95% CI, 1.13-2.02, p=0.005, **Figure 2A**). At the time of analysis, the median OS was not reached. However, for the first-line treatment, PyroH resulted in a median PFS of 14.46 months, contrasting with 22.90 months for HP (HR 1.85, 95% CI 0.86–3.94, p=0.057, **Figure 2B**). For the treatments post second-line, PyroH had a median PFS of 8.67 months while HP had a median PFS of 7.92 months (HR 0.82, 95% CI 0.57–1.18, p=0.286, **Figure 2C**).

The study further evaluated the efficacy of PyroH and HP in conjunction with or without taxane. Without taxane, PyroH patients achieved a median PFS of 10.12 months, while HP patients had a median PFS of 8.15 months (HR 0.58, 95% CI 0.33–1.00, p=0.017, **Figure 2D**). In contrast, with taxane use, PyroH patients had a median PFS of 7.62 months, while HP patients had a median PFS of 19.48 months (HR 2.29, 95% CI 1.30–4.05, p<0.001, **Figure 2E**).

No significant difference was found in the effectiveness of PyroH and HP for patients who developed brain metastases, while PyroH demonstrated a marginally longer median PFS than HP (9.03 vs. 8.15 months, HR 0.99, 95% CI 0.56–1.76, *p*=0.976, **Figure 2F**). However, individuals who did not have brain metastases experienced a median PFS of 10.02 months with PyroH treatment, whereas those who received HP treatment had a median PFS of 14.95 months (HR 1.65, 95% CI 1.16–2.34, p=0.004, **Figure 2E**).

**Figure 3:**
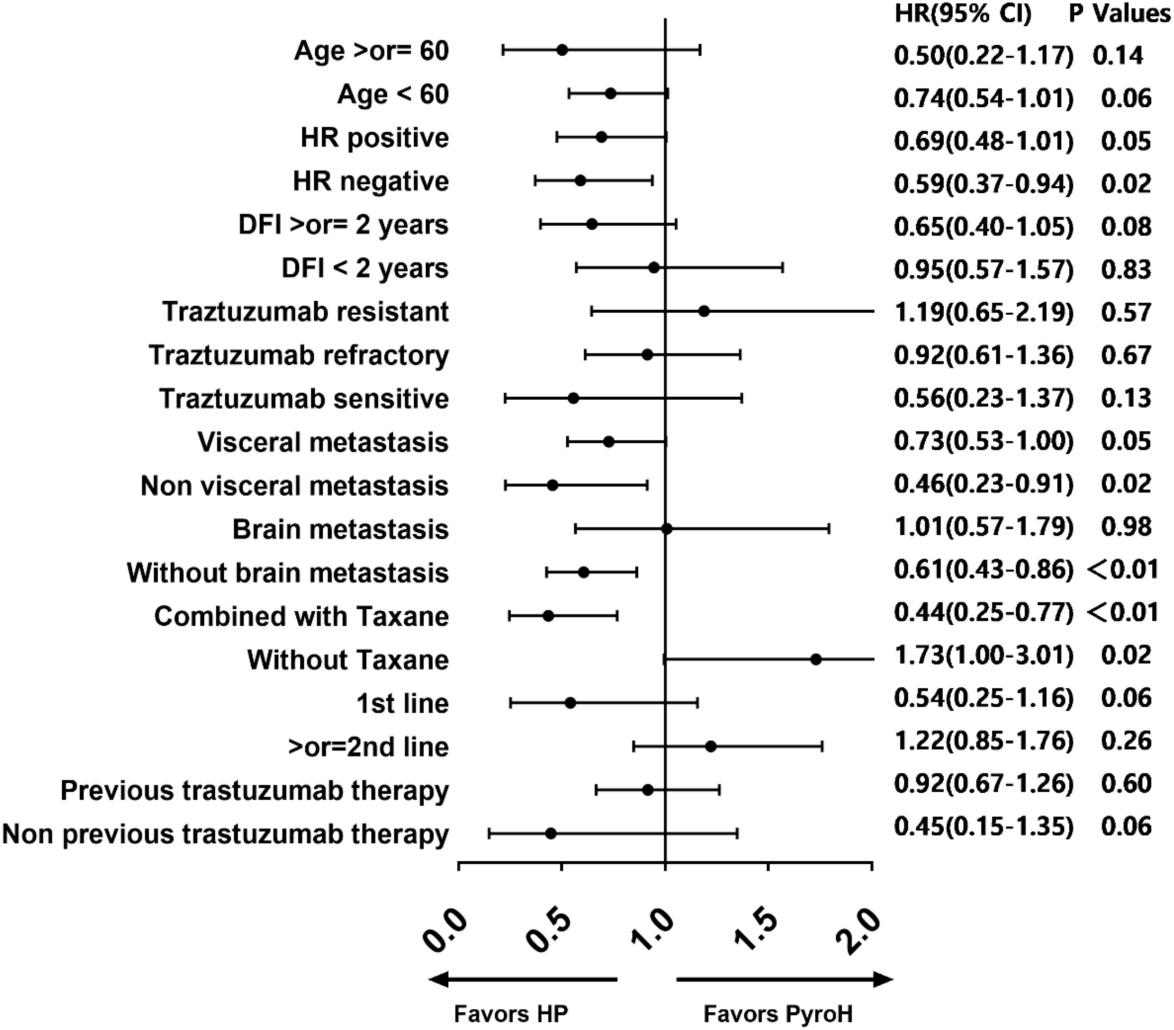
Forest plot for subgroup analysis.

Based on the forest diagram, HP exhibited better efficacy than PyroH in the subgroup of patients with HR-negative, non-visceral metastases, non-brain metastases, and those who received concomitant taxane therapy (**Figure 3**). In the subset of patients who were resistant to traztuzumab and had undergone at least two treatment lines, there was a noticeable inclination towards improved PFS with PyroH compared to HP, although no significant disparity was found. In the subgroup without the combination of taxane, PyroH demonstrated significantly better efficacy than HP. In first-line treatment with PyroH or HP, the 95% CIs of HR for all subgroups suggest no statistical difference in PFS between the HP and PyroH cohorts (**Supplementary Figure 1**). However, HP is more effective in the traztuzumab-sensitive, non-visceral metastasis, and non-brain metastasis subgroups in the first-line treatment (p<0.05). The HR value for the traztuzumab-resistant patients in the first line is greater than 1, which may suggest that PyroH may be more effective, although the 95% confidence interval of HR shows no statistical significance.

**Table 2** contains the outcomes of univariate and multivariate Cox regression analyses for factors linked to PFS. In univariate analysis, several factors were significantly associated with PFS, including treatment group (HR 0.659, 95%CI 0.493-0.882, *p*=0.005), DFI (HR 0.642, 95% CI 0.453-0.908, *p*=0.012), number of metastatic sites (HR 0.527, 95% CI 0.392-0.708, *p*<0.001), lines of advanced systematic therapy (HR 0.457, 95% CI 0.342-0.612, *p*<0.001), trastuzumab resistance status (HR 2.337, 95% CI 1.609-3.394, *p*<0.001), prior exposure to trastuzumab (HR 0.420, 95% CI 0.281-0.629, P<0.001) and combination with taxane (HR 1.637, 95% CI 1.225-2.187, *p*=0.001). In multivariate analysis, number of metastatic sites (HR 0.595, 95% CI 0.388-0.912, *p*=0.017) and lines of advanced systematic therapy (HR 0.564, 95% CI 0.368-0.863, *p*=0.008) remained significant PFS predictors, while the treatment group did not independently predict PFS in HER2+ MBC (HR 1.134, 95% CI 0.710-1.811, p=0.598).

**Table 2:** Univariate and multivariate Cox regression analysis of factors associated with progression-free survival.

Characteristic	HR (95% CI)	Univariate Cox analysis *P*-value	HR (95% CI)	Multivariate Cox analysis *P*-value
Treatment group (HP vs. PyroH)	0.659(0.493-0.882)	0.005	1.134(0.710-1.811)	0.598
Age group (<60 vs. ≥60)	1.229(0.785-1.925)	0.367	0.943(0.534-1.665)	0.840
Hormone receptor status (HR+ vs. HR-)	0.865(0.643-1.163)	0.337	0.868(0.585-1.287)	0.481
DFI (<2year vs. ≥2year)	1.558(1.101-2.205)	0.012	1.342(0.911-1.978)	0.137
Number of metastatic sites (≤2 vs. >2)	0.527(0.392-0.708)	<0.001	0.595(0.388-0.912)	0.017
Visceral metastases (no vs. yes)	0.869(0.601-1.256)	0.454	1.126(0.698-1.816)	0.628
Brain metastases (no vs. yes)	0.800(0.582-1.100)	0.170	0.947(0.607-1.477)	0.810
Lines of advanced systematic therapy (1-2 vs. ≥3)	0.457(0.342-0.612)	<0.001	0.564(0.368-0.863)	0.008
Trastuzumab resistance status (resistance/refractoriness vs. sensitivity)	2.337(1.609-3.394)	<0.001	1.102(0.443-2.744)	0.834
Prior exposure to trastuzumab (no vs. yes)	0.420(0.281-0.629)	<0.001	0.721(0.258-2.011)	0.532
Combination with taxane (no vs. yes)	1.637(1.225-2.187)	0.001	1.117(0.700-1.783)	0.643

HR, Hormone receptor; DFI, Disease free interval.

**Supplementary Table 4** provides the univariate and multivariate Cox regression analysis of factors associated with PFS in first-line systemic treatment. Univariate analysis indicated that hormone receptor status (HR 1.880, 95% CI 1.018-3.473, p=0.044) and number of metastatic sites (HR 0.424, 95% CI 0.235-0.764, p=0.004) were significantly associated with PFS. In multivariate analysis, the number of metastatic sites (HR 0.220, 95% CI 0.055-0.880, p=0.032), trastuzumab resistance status (HR 0.051, 95% CI 0.005-0.554, p=0.014) and prior exposure to trastuzumab (HR 0.177, 95% CI 0.045-0.702, p=0.014) emerged as independent prognostic factors for PFS in the first-line systemic treatment.

### Safety

Diarrhea was the most common grade 3/4 adverse event in the PyroH group, with 34.3% of patients experiencing it, significantly higher than that in the HP group (3.0%, p<0.001) (**Table 3**). However, compared to the HP group, the PyroH group exhibited a lower occurrence of neutropenia (3.0% vs. 14.5%, p<0.001). Palmar-plantar erythrodysesthesia syndrome (6.0% vs. 0.0%, p=0.001) and increased AST or/and ALT (0.6% vs. 7.0%, p=0.003) were also more common in the PyroH group. However, no notable disparities were observed between the two groups regarding the occurrence of other unfavorable incidents. Overall, the incidence of adverse events in PyroH group was not significantly different compared to the HP group (122/161 vs. 119/172, p=0.222). The most frequent adverse effect in PyroH group, diarrhea, typically occurred during the first two weeks of treatment commencement and could be managed by either reducing the dosage of pyrotinib or using loperamide.

**Table 3:** Adverse Events (grade 3/4).

Adverse Events (grade 3/4)	Pyrotinib plus Trastuzumab (PyroH) group (N=166) n (%)	Pertuzumab plus Trastuzumab (HP) group (N=172) n (%)	p-value
Diarrhea	57 (34.3)	17 (3.0)	<0.001
Neutropenia	5 (3.0)	25 (14.5)	<0.001
Leukopenia	6 (3.6)	16 (9.3)	0.480
Thrombocytopenia	2 (1.2)	8 (4.7)	0.106
Anemia	0 (0.0)	6 (3.5)	0.030
Fatigue	5 (3.0)	8 (4.7)	0.576
Vomiting	9 (5.4)	4 (2.3)	0.160
Nausea	5 (3.0)	2 (1.2)	0.270
Decreased Appetite	2 (1.2)	2 (1.2)	1.000
Peripheral neurotoxicity	3 (1.8)	7 (4.1)	0.339
Palmar-plantar erythrodysesthesia syndrome	10 (6.0)	0 (0.0)	0.001
AST or/and ALT increased	1 (0.6)	12 (7.0)	0.003
Rash	5 (3.0)	4 (2.3)	0.744
Stomatitis	5 (3.0)	3 (1.7)	0.490
Weight Loss	3 (1.8)	0	0.112
Hypokalaemia	1 (0.6)	2 (1.2)	1.000
Upper Respiratory Tract Infection	0 (0.0)	1 (0.6)	1.000
Blood Alkaline Phosphatase Increased	1 (0.6)	0 (0.0)	0.483
Blood Bilirubin Increased	1 (0.6)	1 (0.6)	1.000
γ-glutamyltransferase Increased	1 (0.6)	0 (0.0)	0.483
Pigmentation Disorder	0 (0.0)	1 (0.6)	1.000

## Discussion

This study conducted a direct comparison of the clinical practice of PyroH and HP in patients with HER2+ MBC in the real-world. As well as we have known, this study is the first research to directly compare PyroH and HP in the management of MBC. Even though the HP group exhibited a considerably greater mPFS compared to the PyroH group overall, the treatment group does not serve as a standalone predictor of PFS in the multivariate analysis. According to our study, PyroH and HP have comparable effectiveness in first-line and subsequent advanced therapies, respectively. However, their efficacy can be influenced by the treatment lines, the addition of taxane, and the presence of brain metastasis. When taxane is not available or when there are brain metastases, PyroH could be considered as a viable choice.

Prior to December 2022, PyroH was not included as a recommended treatment option for HER2+ MBC in either international or domestic treatment guidelines. In China, the standard second-line therapy for advanced stages involved the combination of pyrotinib with capecitabine. Consequently, the utilization of PyroH in real-world clinical practice was relatively limited. Therefore, this study incorporated data from five institutions, only encompassing a cohort of 161 patients treated with PyroH.

Our analysis reveals that in a real-world context, a mere 24 patients received PyroH as a first-line treatment. Notably, PyroH was more commonly administered to older patients, those with a disease-free interval of less than two years, and individuals who had previously undergone trastuzumab therapy. Furthermore, the incidence of brain metastases was numerically higher in the PyroH group compared to the group receiving HP. Nevertheless, PyroH did not demonstrate a statistically noteworthy difference in median PFS when compared to HP in first-line therapy, despite indicating a tendency towards a reduced PFS in the PyroH cohort. Efficacy data of HP regimen is consistent with previous studies (3, 10) (median PFS of 20.7 months in PERUSE; 18.7 months in CLEOPATRA). The PFS of PyroH group in the first-line therapy is comparable to a pooled analysis (11) of combined 145 patients receiving pyrotinib combined with capecitabine showing a median PFS of 12.4 months, but is much shorter than the median PFS of 24.3 months observed in the PHILA trail (12). The difference could be due to the fact that, in our research, a higher proportion of individuals in the PyroH group had previously received trastuzumab treatment, and some of the patients treated with PyroH did not receive taxane administration but instead received a combination of capecitabine or vinorelbine; furthermore, in real-world settings, the circumstances are more intricate, and patients may exhibit inadequate drug compliance. Future investigations involving a greater population and extended follow-up duration will be crucial to substantiate these results and discern the potential advantages of PyroH among particular patient cohorts.

With the increasing prevalence and insurance coverage of trastuzumab in HER2+ MBC patients, a considerable number of patients receiving trastuzumab in the early stage has emerged. However, there are only about 11% of trastuzumab-treated patients in the CLEOPATRA (3) and PUFFIN (13) studies and subgroup analyses have demonstrated a notably lower PFS benefit for HP regimens in trastuzumab-treated patients as opposed to trastuzumab-naïve patients. Subgroup analysis from the PERUSE study (10) has once again implied a reduced efficacy of advanced first-line treatment of HP dual-targeted regimens in trastuzumab-treated patients (median PFS: 15.4 months vs. 23.4 months). Our investigation revealed a greater percentage of subjects in the PyroH cohort (90.0%) had received prior trastuzumab treatment when compared to the HP cohort (58.1%). In the first-line treatment, a higher percentage of patients in the PyroH cohort (50.0%) previously underwent trastuzumab treatment compared with 22.0% in the HP group, which may reflect the clinical practice and prognosis of dual-HER2-targeted therapy in the real-world post-trastuzumab era.

The study findings also suggest that the majority of patients in both the PyroH and HP groups had trastuzumab refractoriness rather than trastuzumab resistance. Furthermore, there existed a notable contrast in the distribution of trastuzumab resistance status among the two cohorts, as the HP group displayed a greater percentage of trastuzumab-sensitive individuals while the PyroH group exhibited a higher proportion of trastuzumab-resistance and refractoriness. This finding implies that PyroH may be a frequently employed therapeutic modality for patients who have undergone previous trastuzumab administration. Conversely, the HP group displayed a higher incidence of trastuzumab-sensitive patients and a lower prevalence of previously treated individuals, indicating that HP is a more customary first-line therapy for HER2+ MBC. These findings may have contributed to the results that PyroH had a significantly shorter median PFS compared to HP overall. However, when comparing first-line systemic treatment and post second-line of systemic treatment, PyroH had a shorter median PFS compared to HP for first-line treatment but similar median PFS for post second-line systemic treatment. These results suggest that patient’s previous treatment history and trastuzumab resistance status may play a significant role in the choice of treatment. Additional research is required to validate these discoveries and ascertain the most effective therapeutic approaches for patients with HER2+ MBC.

We also compared the clinical practices of administering PyroH and HP in HER2+ MBC, observing that PyroH was more frequently paired with orally-administered chemotherapeutic agents, including capecitabine, vinorelbine, and VP-16, while HP was typically administered in conjunction with taxane-based chemotherapeutic agents. This dissimilarity in treatment regimen was statistically significant. The PyroH group had a greater proportion of patients who did not undergo chemotherapy compared to the HP group. In first-line therapy, both HP and PyroH groups predominantly utilized taxane as chemotherapy combination, with a certain number of PyroH patients opting for either capecitabine or vinorelbine. Notably, PyroH exhibited better efficacy than HP when taxane was not utilized in the treatment. However, when taxane was employed, HP demonstrated a better PFS than PyroH. The study suggests that distinct chemotherapy regimens were used for administering PyroH and HP in clinical practice, which may have an impact on their efficacy. The study also provides critical insights into the influence of taxane-based chemotherapy on the efficacy of PyroH and HP. Considering taxane resistance is still common in breast cancer (14), PyroH may be a better option when taxane is not used, as it exhibited better efficacy than HP in this scenario. The feasibility of giving priority to PyroH over HP regimens in the taxane-resistant population is a matter worth more investigation.

In patients with HER2+ MBC, brain metastasis affects 30%-50% of cases (15). In the PHENIX study (5), 31 patients with brain metastases were included, and the group that received the combination of pyrotinib and capecitabine had a reported median PFS of 6.9 months. Several retrospective real-world studies demonstrated the efficacy of pyrotinib against brain metastases as well, showing a median PFS ranging from 6.3-8.8 months (16–18). Our study revealed significant differences in the proportion of individuals with brain metastases between the PyroH and HP groups, with a greater proportion of patients in the PyroH group having brain metastases. PyroH group exhibited a longer median PFS than the HP group in patients with brain metastases, although no statistically significant difference was observed. Conversely, for patients without brain metastases, PyroH treatment resulted in a shorter median PFS compared to HP. Our study indicates that PyroH could potentially be a feasible therapeutic choice for individuals suffering from HER2+ MBC with brain metastases, which may consistent with previous investigation (7, 19). To validate these findings, additional research with bigger sample sizes is still required. Moreover, our study highlights the importance of considering patient characteristics, such as brain metastases, when selecting the optimal treatment for HER2+ MBC.

According to the safety analysis, both treatment plans were well-tolerated. Diarrhea, as a grade 3/4 adverse event, was reported in 34.3% in PyroH group, a rate slightly elevated compared to 15.4-30.8% in a previous study of pyrotinib (5, 19). This occurrence was notably higher compared to the 3.0% incidence in the HP group. However, neutropenia was less prevalent in the PyroH group. Although the PyroH group experienced a higher prevalence of diarrhea compared to the HP group, the occurrence of severe adverse events of grade 3/4 was generally similar in both groups. Effective management of adverse events, such as early intervention for diarrhea, is crucial for ensuring patient safety and treatment efficacy. Diarrhea could be managed using standard loperamide or montmorillonite interventions, and if necessary, dosage adjustments were implemented.

This study carries certain limitations worth noting. The study was retrospective in nature. There were imbalanced patient baseline characteristics between the PyroH and HP groups, which highlights the need for a prospective randomized controlled trial. Differences in the number of treatment lines, metastasis, trastuzumab resistance, and compatible chemotherapy drugs between the PyroH and HP groups could have affected the relative efficacy of PyroH and HP.

It’s widely recognized that the combined therapy of trastuzumab, pertuzumab, and taxane is a recommended first-line therapy for HER2+ MBC patients (10). For patients who have progressed after trastuzumab-based treatment, trastuzumab deruxtecan (DS8201) (20) and tucatinib combined with trastuzumab and capecitabine (21) have emerged as preferred regimens. Unfortunately, due to limited availability and economic factors, DS8201 and tucatinib are not commonly used in clinical practice in China nowadays. Yet, hope is found in pyrotinib, an orally administered TKI approved for use in China, with several trials exploring its potential in conjunction with trastuzumab for initial MBC treatment (12), as well as in neo-adjuvant settings for early-stage breast cancer (22, 23). As far as we know, our study is the first and most extensive investigation of its type, providing real-world evidence in the comparison between PyroH and HP. Furthermore, the comparison of treatment patterns and safety data for PyroH and HP in real-world clinical practice could provide a theoretical foundation for clinicians.

In conclusion, PyroH and HP cohorts had similar PFS in the second line and subsequent treatment. The efficacy of these treatments may have been impacted by the treatment line, the inclusion of taxane, and the presence of brain metastases. Both regimens were well-tolerated in terms of toxicity. Hence, PyroH may prove to be a rational choice when taxane is not administered or when brain metastases are present.

## Data availability statement

The raw data supporting the conclusions of this article will be made available by the authors, without undue reservation.

## Ethics statement

The studies involving humans were approved by the Institutional Review Board of Fudan University Cancer Hospital (SCCIRB), 1812195-6. The studies were conducted in accordance with the local legislation and institutional requirements. The ethics committee/institutional review board waived the requirement of written informed consent for participation from the participants or the participants’ legal guardians/next of kin because the investigation is retrospective.

## Author contributions

SY: Data curation, Formal analysis, Investigation, Methodology, Writing – original draft. YX: Conceptualization, Data curation, Writing – original draft, Writing – review & editing. DS: Data curation, Writing – review & editing. TL: Data curation, Writing – review & editing. PY: Data curation, Writing – review & editing. FX: Data curation, Writing – review & editing. BW: Conceptualization, Funding acquisition, Writing – review & editing.
