# Supplementary material for: Comparing pyrotinib with trastuzumab and pertuzumab with trastuzumab for HER2-positive metastatic breast cancer: a retrospective, multicenter analysis
Source: Front Endocrinol (Lausanne). 2023 Dec 11;14:1325540. doi: 10.3389/fendo.2023.1325540 (PMC10750407; doi:10.3389/fendo.2023.1325540)

Supplementary Figure 1. Forest plot for subgroup analysis in the first-line systemic treatment for MBC.

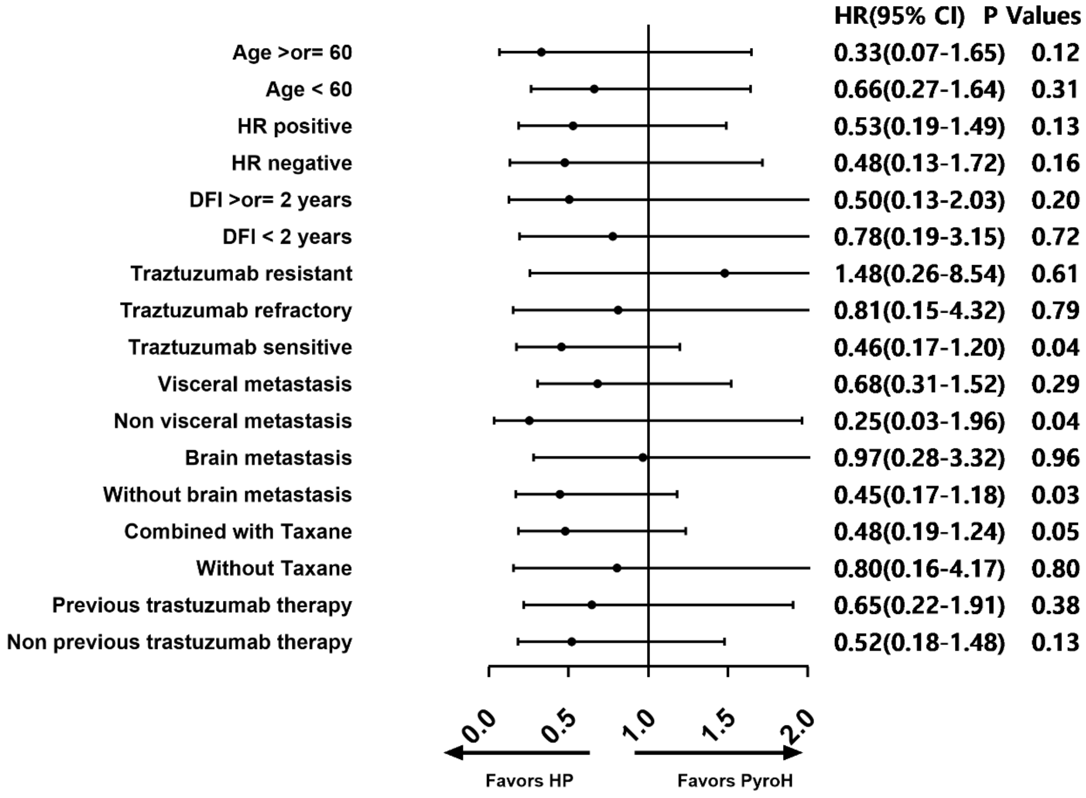

Supplement: Supplementary file 1 [file Image_1.pdf]
