# Supplementary material for: Comparing pyrotinib with trastuzumab and pertuzumab with trastuzumab for HER2-positive metastatic breast cancer: a retrospective, multicenter analysis
Source: Front Endocrinol (Lausanne). 2023 Dec 11;14:1325540. doi: 10.3389/fendo.2023.1325540 (PMC10750407; doi:10.3389/fendo.2023.1325540)
Supplement: Supplementary file 2 [file Table_1.docx]

Supplementary table 1. Baseline characteristics of patients receiving PyroH or HP at the first line of advanced systemic treatment.

|  | | Pyrotinib plus trastuzumab (PyroH) group  N=24  n (%) | Pertuzumab plus trastuzumab (HP) group  N=100  n (%) | p-value |
| --- | --- | --- | --- | --- |
| Age, years | | 54 (39-83) | 49 (26-71) | 0.004 |
| Eastern Cooperative Oncology Group performance status | | | | 0.131 |
|  | 0 | 3 (12.5) | 18 (18.0) |  |
|  | 1 | 17 (70.8) | 75 (75.0) |  |
|  | 2 | 3 (12.5) | 3 (3.0) |  |
| Hormone receptor status | | | | 0.851 |
|  | Estrogen receptor or progesterone receptor positive | 12 (50.0) | 50 (50.0) |  |
|  | Estrogen receptor and progesterone receptor negative | 11 (45.8) | 50 (50.0) |  |
| Pathological grading | | | | 0.093 |
|  | I | 2 (8.3) | 0 (0.0) |  |
|  | II | 8 (33.3) | 29 (90.0) |  |
|  | III | 10 (41.7) | 30 (90.0) |  |
| Disease-free interval | |  |  | 0.031 |
|  | ＜2 years | 9 (37.5) | 11 (11.0) |  |
|  | ≥2 years | 8 (33.3) | 38 (38.0) |  |
|  | de novo stage IV breast cancer | 7 (29.2) | 50 (50.0) | 0.069 |
| Number of metastatic sites | |  |  | 0.210 |
|  | 1 | 8 (33.3) | 36 (36.0) |  |
|  | 2 | 3 (12.5) | 27 (27.0) |  |
|  | ≥3 | 13 (54.2) | 37 (37.0) |  |
| Metastatic sites at screening | |  |  |  |
|  | Visceral | 19 (79.2) | 73 (73.0) | 0.613 |
|  | Brain | 7 (29.2) | 12 (12.0) | 0.055 |
| Trastuzumab resistance status | |  |  | 0.078 |
|  | Resistance | 4 (16.7) | 3 (3.0) |  |
|  | Refractoriness | 4 (16.7) | 16 (16.0) |  |
|  | Sensitivity | 16 (66.7) | 80 (80.0) |  |
| Previous trastuzumab therapy | |  |  | 0.009 |
|  | Yes | 12 (50.0) | 22 (22.0) |  |
|  | No | 11 (45.8) | 74 (74.0) |  |
| Combined with taxane | |  |  | ＜0.001 |
|  | Yes | 13 (54.2) | 94 (94.0) |  |
|  | No | 11 (45.8) | 6 (6.0) |  |
| Combined with capecitabine or vinorelbine or VP-16 | | | | 0.179 |
|  | Yes | 8 (33.3) | 20 (20.0) |  |
|  | No | 16 (66.7) | 80 (80.0) |  |

Supplementary table 2. Chemotherapy combined with PyroH or HP for MBC.

|  | Pyrotinib plus trastuzumab group (PyroH)  N=161 n (%) | Pertuzumab plus trastuzumab group (HP)  N=172 n (%) |
| --- | --- | --- |
| Taxane | 34 (21.1) | 110 (64.0) |
| Capecitabine | 48 (29.8) | 6 (3.5) |
| Vinorelbine | 28 (17.4) | 12 (7.0) |
| Gemcitabine | 6 (3.7) | 0 (0.0) |
| Taxane + Platinum | 3 (1.9) | 22 (12.8) |
| Taxane + Capecitabine | 2 (1.2) | 4 (2.3) |
| Gemcitabine + Platinum | 0 (0.0) | 4 (2.3) |
| Eribulin | 5 (3.1) | 2 (1.1) |
| Others* | 14 (8.7) | 4 (2.3) |
| Without chemotherapy | 21 (13.0) | 8 (4.7) |

*In PyroH group, it included VP-16, doxorubicin, pirarubicin + cyclophosphamide, vinorelbine + capecitabine, capecitabine sequenced by VP-16, gemcitabine + capecitabine, capecitabine or vinorelbine sequenced by paclitaxel, capecitabine sequenced with vinorelbine, docetaxel and loplatin sequenced by doxorubicin, capecitabine sequenced by gemcitabine, gemcitabine or vinorelbine sequenced by eriburin; in HP gourp, it included vinorelbine + capecitabine, vinorelbine sequenced by eriburin

#included endocrine therapy

Supplementary table 3. Chemotherapy combined with PyroH or HP at the first line systemic therapy in MBC.

|  | Pyrotinib plus trastuzumab group (PyroH)  N=24 n (%) | Pertuzumab plus trastuzumab group (HP)  N=100 n (%) |
| --- | --- | --- |
| Taxane | 12 (50.0) | 76(76.0) |
| Capecitabine | 5 (20.8) | 0(0.0) |
| Vinorelbine | 2 (8.3) | 4(4.0) |
| Taxane + Platinum | 1 (4.2) | 15(15.0) |
| Taxane + Capecitabine | 1 (4.2) | 4(4.0) |
| Anthracycline + cyclophosphamide | 1 (4.2) | 0(0.0) |
| Without chemotherapy | 2 (8.3) | 1(1.0) |

Supplementary table 4. Univariate and multivariate Cox regression analysis of factors associated with progression-free survival in the first-line systemic treatment.

| **Characteristic** | **HR (95% CI)** | **Univariate Cox analysis *P*-value** | **HR (95% CI)** | **Multivariate Cox analysis *P*-value** |
| --- | --- | --- | --- | --- |
| Treatment group (HP vs. PyroH) | 0.540(0.284-1.029) | 0.061 | 1.033(0.249-4.278) | 0.964 |
| Age group (<60 vs. ≥60) | 0.884(0.385-2.029) | 0.770 | 0.126(0.017-0.940) | 0.043 |
| Hormone receptor status (HR+ vs. HR-) | 1.880(1.018-3.473) | 0.044 | 0.903(0.236-3.452) | 0.881 |
| DFI (≥2year vs. ＜2year) | 1.056(0.464-2.404) | 0.896 | 1.655(0.384-7.129) | 0.499 |
| Number of metastatic sites (≤2 vs. >2) | 0.424(0.235-0.764) | 0.004 | 0.220(0.055-0.880) | 0.032 |
| Visceral metastases (no vs. yes) | 0.613(0.285-1.318) | 0.210 | 0.787(0.080-7.716) | 0.837 |
| Brain metastases (no vs. yes) | 0.634(0.321-1.254) | 0.191 | 2.962(0.469-18.727) | 0.248 |
| Trastuzumab resistance status (resistance/refractoriness vs. sensitivity) | 1.045(0.551-1.984) | 0.892 | 0.051(0.005-0.554) | 0.014 |
| Prior exposure to trastuzumab (no vs. yes) | 0.657(0.368-1.175) | 0.157 | 0.177(0.045-0.702) | 0.014 |
| Combination with taxane (no vs. yes) | 1.459(0.614-3.466) | 0.392 | 0.397(0.044-3.593) | 0.411 |

HR, Hormone receptor; DFI, Disease free interval
